# Knocking down tumor suppressor gene PTPRG enhances axonal regeneration of dorsal root ganglion neurons

**DOI:** 10.3389/fcell.2025.1698864

**Published:** 2025-11-13

**Authors:** Qian Zhao, Lan Zhang, Peng Yang, Jingjing Li, Sheng Yi

**Affiliations:** 1 Key Laboratory of Neuroregeneration of Jiangsu and Ministry of Education, Co-innovation Center of Neuroregeneration, NMPA Key Laboratory for Research and Evaluation of Tissue Engineering Technology Products, Nantong University, Nantong, Jiangsu, China; 2 Department of Emergency, Affiliated Hospital of Nantong University, Nantong University, Nantong, Jiangsu, China

**Keywords:** nerve injury, axonal regeneration, dorsal root ganglion, tumor suppressor gene, PTPRG

## Abstract

**Introduction:**

Nerve injuries severely impair quality of life, with the limited axonal regenerative capacity in mammals hindering functional recovery. Dorsal root ganglion (DRG) neurons serve as an essential model for the identification of axonal regeneration regulators as their peripheral axonal branches are regeneration permissive after axotomy while their central axonal branches are regeneration incompetent.

**Methods:**

In this study, we analyze transcriptional profiling of rat DRGs in response to peripheral and central axonal injuries. We use siRNA-mediated silencing of the tumor suppressor gene PTPRG (protein tyrosine phosphatase receptor type G) in cultured DRG neurons and explants to assess its role in neurite outgrowth and axonal regeneration. RNA sequencing is used to identify associated pathways and gene expression changes.

**Results:**

We find that the expression of PTPRG is reduced after injury to peripheral axonal branches but increased after injury to central axonal branches. In cultured DRG neurons and DRG explants, siRNA-mediated silencing of PTPRG leads to boosted neurite outgrowth and enhanced sensory axonal regeneration. Sequencing data show that PTPRG knockdown is associated with the activation of metabolism-related pathways and altered expression of the transcription factor-coding gene prospero-related homeobox 1 (PROX1).

**Discussion:**

These findings identify PTPRG as a novel negative regulator of axonal regeneration, expanding the repertoire of molecules that can be manipulated to improve functional recovery after nerve injuries.

## Introduction

Nerve injuries, encompassing damage to the central and peripheral nervous systems, are a major public health burden with inadequate functional rehabilitation. Affected patients typically experience motor dysfunction (weakness or paralysis), sensory loss, and chronic neuropathic pain, all of which substantially compromise patients’ quality of life (Gu et al., 2024; Zhao L. et al., 2024). Axons are elongated projections of neurons that serve as the main transmission pathways of the nervous system. The functional regeneration of injured axons is essential for meaningful neurological recovery after nerve injury and in many neurological disorders (Hu et al., 2021; Yang et al., 2021). In many invertebrates and non-mammalian vertebrates, axotomy triggers a cascade of regeneration-promoting molecular and cellular responses in neurons and severed axon stumps are capable of regrowing and functionally reconnecting with their targets (He and Jin, 2016). However, in the mammalian nervous system, the regenerative capacity of axons is limited, presenting a severe barrier to clinical functional restoration (Zheng and Tuszynski, 2023).

Enhancing neurons’ intrinsic growth competency is a key approach for successful axonal regeneration and functional repair. Advances in regenerative neurobiology have firmly established the tumor suppressor gene PTEN (phosphatase and tensin homolog) as a master regulator of neuronal behavior, specifically the intrinsic axonal growth potential (Park et al., 2008; Liu et al., 2022). In the central nervous system, deletion of PTEN, via enhancing the activity of the mammalian target of rapamycin (mTOR), boosts the regenerative capacity of retinal ganglion cell axons and corticospinal tract axons, following optic nerve injury and spinal cord injury, respectively (Park et al., 2008; Liu et al., 2010; Stewart et al., 2023). In the peripheral nervous system, PTEN inhibition activates phosphatidylinositol 3-kinase (PI3K)/protein kinase B (PKB/AKT) signaling and supports the outgrowth of axons, especially IB4-positive neuronal axons, following sciatic nerve injury (Christie et al., 2010; Zhou et al., 2020). Strategies targeting PTEN, as well as other intrinsic regenerative regulators, represent a critical therapeutic avenue to enable sustained long-distance axonal elongation and complete functional recovery (Sun et al., 2011).

The intrinsic growth capacity of injured peripheral nerves is remarkably higher than that of central nerves, although severe peripheral nerve injuries, such as long nerve gaps and/or prolonged denervation, can still elicit permanent neurological disorders (Mahar and Cavalli, 2018; Zhang et al., 2025). Dorsal root ganglion (DRG) neurons are pseudo-unipolar sensory neurons whose stem axons bifurcate to regeneration-permissive peripheral axonal branches and regeneration-incompetent central axonal branches. This distinctive morphology of enables comparative studies of regeneration-permissive versus non-permissive injury responses within the same neuronal somata and benefits the discovery of essential regulators of axon regeneration (Zhao Q. et al., 2024; Costa et al., 2025).

In the present study, we performed a comprehensive transcriptomic analysis of rat DRGs using our laboratory’s gene profiling dataset (Cao et al., 2022), and explored transcriptional alterations following sciatic nerve injury-induced peripheral axonal injury and dorsal root injury-induced-central axonal injury. We specifically analyzed genes exhibiting inverse expression patterns in response to peripheral versus central axonal injury. We found that the tumor suppressor gene PTPRG (protein tyrosine phosphatase receptor type G) displays divergent injury responses, with significantly downregulation in DRGs after peripheral axonal injury but noticeably upregulation after central axonal injury. Next, we functionally characterized PTPRG by performing siRNA-mediated knockdown and demonstrated the beneficial roles of PTPRG inhibition in axonal growth. Our study establishes PTPRG as a negative regulator of axonal regeneration, providing a novel therapeutic target for the treatment of nerve injuries.

## Materials and methods

### Animals and surgical procedures

Specific-pathogen-free Sprague-Dawley (SD) rats were obtained from the Experiment Animal Center of Nantong University. Animal experiments were approved by the Ethics Committees of Nantong University. Surgical procedures were conducted in accordance with the Institutional Animal Care guidelines of Nantong University. Neonatal SD rats were used for DRG harvesting. Adult male SD rat weighing 180–220 g were subjected to DRG neuron isolation as well as nerve injury or sham surgery.

Sciatic nerve injury and dorsal root injury were performed as previously reported (Cao et al., 2022; Chen et al., 2023). Briefly, for sciatic nerve injury, rats were deeply anesthetized with an intraperitoneal injection of 40 mg/kg sodium pentobarbital. The biceps femoris and the gluteus superficialis were bluntly dissected, and the sciatic nerve was exposed. The sciatic nerve was completely transected with microscissors or crushed for 30 s with forceps. For dorsal root injury, the dura mater was removed with fine forceps and the exposed dorsal root was transected with microscissors. Sham-operated rats underwent exposure of the sciatic nerve or the dorsal root without axotomy or crush injury were used as surgery controls. Rats were euthanized via CO_2_ asphyxiation.

### RNA sequencing and bioinformatic analysis

RNA sequencing data of rat L4-L5 DRGs collected at 1 day following sciatic nerve transection versus sham and dorsal root transection versus sham have been published and deposited in the Genome Sequence Archive database with accession number CRA006070 (Cao et al., 2022). RNA sequencing of primary cultured DRG neurons transfected with siRNA against PTPRG or negative control siRNA was performed on Illumina HiSeq^TM^ 4,000 by Genedenovo Biotechnology Co., Ltd. (Guangzhou, Guangdong, China). Sequencing data were deposited in the Genome Sequence Archive database with accession number CRA024297. Briefly, total RNA was isolated from collected tissues or cells using TRIzol reagent (Invitrogen, Carlsbad, CA, United States). After the removal of rRNAs, mRNAs were enriched, fragmented, and reverse transcribed to cDNAs with random primers. Second-strand cDNAs were synthesized, end-repaired, poly(A)-tailed, ligated to Illumina sequencing adapters, and then digested. The digested products then underwent size selection, amplification, and RNA sequencing.

Differentially expressed genes were detected using edgeR with the parameters of an absolute fold change greater than or equal to 2 and a false discovery rate below 0.05. These differentially expressed genes were mapped to Gene Ontology (GO) terms and Kyoto Encyclopedia of Genes and Genomes (KEGG) pathways. Gene signatures were further explored using Gene Set Enrichment Analysis (GSEA). Enrichment analysis of GO, KEGG, and GSEA, as well as the generation of heatmaps, were carried out using Omicsmart, a dynamic real-time interactive online platform for data analysis (http://www.omicsmart.com). Potential transcriptional downstream targets were systematically identified by employing multiple transcription factor databases, including knockTF, FIMO, and JASPAR.

### Quantitative real-time polymerase chain reaction (qRT-PCR) analysis

Total RNA extracted from DRGs or neurons was treated with amplification-grade DNase I (Thermo Fisher Scientific, Waltham, MA, United States) and reverse transcribed to cDNA using the HiScript III RT SuperMix for qPCR (Vazyme, Nanjing, China). cDNA was amplified in a 10-μL reaction containing SYBR Mix (Vazyme) on an ABI StepOne system (Applied Biosystems, Foster City, CA, United States). The relative mRNA abundance of target gene PTPRG was normalized against the reference gene GAPDH and quantitated using the comparative 2^−ΔΔCt^ method. The following primer sequences were used: PTPRG-F: 5′-GCATATCAGGACACAGCGGA-3′, and PTPRG-R: 5′-TCCCGAGAATGGCTTCCAAC-3’; GAPDH-F: 5′-ACAGCAACAGGGTGGTGGAC-3′ and GAPDH-R:5′-TTTGAGGTGCAGCGAACTT-3’.

### Primary culture of DRG neurons

DRGs isolated from adult SD rats were minced into small pieces, incubated in 3 mg/mL collagenase (Sigma, St. Louis, MO, United States) at 37 °C for 90 min, and digested with 0.25% trypsin at 37 °C for 5 min. After adding serum-containing medium to terminate digestion, cell suspension was filtered, centrifuged to remove the supernatant, and purified with 15% bovine serum albumin. The dissociated DRG neurons were resuspended with Neurobasal medium (Gibco BRL United States, Grand Island, NY, United States) containing 2% B27 supplement (Gibco) and 1% L-glutamine (Beyotime, Shanghai, China). DRG neurons were replated and then seeded onto glass coverslips pre-coated with poly-L-lysine (PLL) or PLL plus purified myelin extracts. Myelin fractions were extracted from adult rat brain tissues using a sucrose density gradient centrifugation protocol as previously described (Ma et al., 2019).

Cultured DRG neurons were fixed with 4% formaldehyde, incubated with a primary antibody against Tuj1 (1:1,000; Abcam, catalog # ab18207, Cambridge, MA, United States) at 4 °C overnight followed by secondary antibody Cy3-conjugated Affinipure Goat Anti-Rabbit IgG (H+L) (1:400; Proteintech, catalog # SA00009-2, Wuhan, China) for 2 h at room temperature. Neuronal cytoskeleton was stained with CytoPainter Phalloidin-iFluor 647 Reagent (1:1,000; Abcam, catalog # ab176759, Cambridge, MA, United States). Immunostaining images were captured on a Zeiss Axio Imager M2 microscope or a Zeiss LSM900 confocal microscope. The total and longest length of neurites in DRG neurons were measured with Neuron J analysis in ImageJ.

### Primary culture of DRG explants

DRGs were collected from neonatal SD rats, cleaned of excess fibers, and plated on glass coverslips pre-coated with PLL. The DRG explants were then incubated with B27 and L-glutamine-containing Neurobasal medium in glass coverslips pre-coated with PLL. DRG explants were fixed with 4% formaldehyde, incubated with a primary antibody against Tuj1 at 4 °C overnight followed by secondary antibody donkey anti-rabbit Alexa Fluor™ 488 (1:400; Invitrogen, Cat # A-21206, Carlsbad, CA, United States) for 2 h at room temperature. Immunostaining images were captured on a Zeiss Axio Imager M2 microscope. The average maximum outgrowth distance was measured as the vector from the explant edge to the axon tip in four directions per explant using the NeuronJ plugin in ImageJ. The length of neurite extension at varying distances from the explant body was quantified using Sholl analysis in ImageJ.

### Transfection assay

Dissociated DRG neurons or extracted DRG explants were transfected with siRNA targeting PTPRG or negative control siRNA using Lipofectamine RNAiMAX transfection reagent (Invitrogen) according to the manufacturer’s instructions. All siRNAs were synthesized by RibiBio Biotechnology Co., Ltd. (Guangzhou, Guangdong, China). The siRNA sequences used were as follows: siRNA-PTPRG-1: 5′-CAGACGACTTTGACAGCTT-3’; siRNA-PTPRG-2: 5′-CTGCACTGGATCCTATTAT-3’; siRNA-PTPRG-3: 5′-CAGTTGCCATCCTGCTGAA-3’; and control siRNA: 5′-GGCUCUAGAAAAGCCUAUGC-3’.

### Terminal deoxynucleotidyl transferase-mediated dUTP nick end labeling (TUNEL) assay

The TUNEL assay was performed as previously described (Xu et al., 2024; Hong et al., 2024). DRG neurons were fixed with 4% formaldehyde, permeabilized with 0.2% Triton X-100, covered by 1× Equilibration Buffer at room temperature for 20 min, and treated with TdT incubation buffer in the TUNEL BrightGreen Apoptosis Detection Kit (Vazyme) at 37 °C for 1 h. DRG neurons were subsequently incubated with the anti-NeuN primary antibody (1:1,000; Abcam, catalog # ab177487) and the corresponding secondary antibody Cy3-conjugated Affinipure Goat Anti-Rabbit IgG (H+L) (1:400; Proteintech). TUNEL and NeuN staining images were captured on a Zeiss Axio Imager M2 microscope. The number of TUNEL-positive neurons was counted with the counting tool in Photoshop CS5.

### Analysis of axonal regeneration following *in vitro* axotomy

*In vitro* axonal regeneration was examined using microfluidic devices with two-compartment chambers (catalog # SND150, Xona 2-compartment SND 150, Xona Microfluidics LLC). DRG neurons were seeded onto the somal compartments to enable their axons to extend into the axonal compartments. A 0.08 MPa vacuum suction was applied to the axonal compartments to transect and remove axons. Injured axons were allowed to grow for an additional 24 h and immunostained with the anti-Tuj1 primary antibody and the corresponding secondary antibody. Immunostaining images were captured on a Zeiss Axio Imager M2 microscope. The average lengths of regenerated axons were quantified using the NeuronJ plugin in ImageJ.

### Single cell sequencing

Single cell RNA sequencing data of male C57BL/6J mouse DRGs have been published and deposited in the GEO database with accession number GSE154659 (Renthal et al., 2020). Gene expression profiles in mouse DRG neurons in the naïve state (designated as 0 day control) and the injured state (at 1 day and 3 days following sciatic nerve crush injury) were evaluated and displayed in a t-distributed stochastic neighbor embedding (tSNE) plot.

### Statistics analysis

Animals and cell cultures were randomly assigned to different groups. Sample size was based on previously established experiments and was described in the figure legends. Statistical analyses were designed using the assumption of normal distribution and performed with GraphPad Prism software using unpaired t-test or ANOVA followed by Dunnett’s or Sidak’s multiple comparisons test. Values were presented as dot plots with individual data points plus bar, representing the mean ± standard error of the mean (SEM). A p-value less than 0.05 was considered to be statistically significant.

## Results

### Sciatic nerve injury and dorsal root injury elicit diverse injury responses in DRGs

**FIGURE 1:**
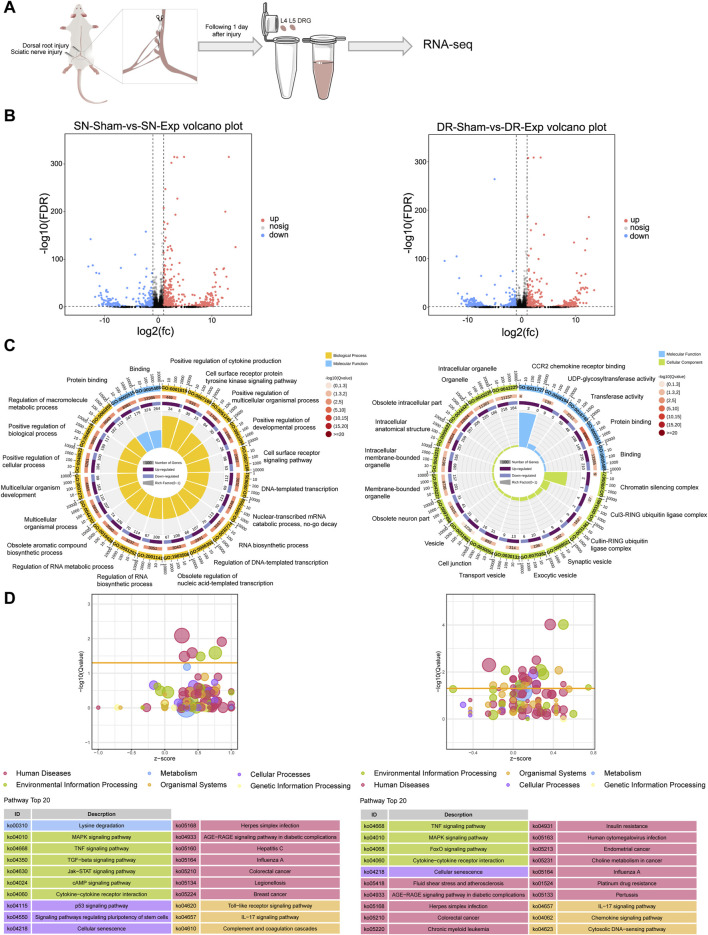
Transcriptomic profiling of rat DRGs following sciatic nerve injury and dorsal root injury. **(A)** Diagrammatic representation of RNA sequencing of rat DRGs after sciatic nerve injury (SN-Exp), dorsal root injury (DR-Exp), or corresponding sham surgery (SN-sham/DR-sham). **(B)** Number of differentially expressed mRNAs in DRGs after sciatic nerve injury or dorsal root injury as compared to the respective sham surgery. **(C)** Enriched GO terms in DRGs after sciatic nerve injury or dorsal root injury. **(D)** Enriched KEGG pathways in DRGs after sciatic nerve injury or dorsal root injury.

We analyzed previously generated RNA sequencing data of rat L4-L5 DRGs at 1 day after injury to either their peripheral axonal branches (via sciatic nerve injury) or their central axonal branches (via dorsal root injury). While prior RNA sequencing studies explored the dynamic changes of circular RNAs, the expression profiles of mRNAs in rat DRGs have not been systematically investigated (Cao et al., 2022). Transcriptomic profiling of rat DRGs revealed significant changes in mRNA expression following both sciatic nerve injury and dorsal root injury (Figure 1A). By comparing mRNA expression levels in rat DRGs after sciatic nerve injury versus sciatic nerve sham surgery, we screened a total of 543 upregulated mRNAs and a total of 362 downregulated mRNAs (Figure 1B). Similarly, we discovered a total of 398 upregulated mRNAs and a total of 317 downregulated mRNAs in rat DRGs after dorsal root injury as compared with dorsal root sham surgery (Figure 1B).

Functional annotation clustering of differentially expressed mRNAs using GO analysis revealed significant enrichment of biological processes related to cytokine (positive regulation of cytokine production), cell signaling pathway (cell surface receptor protein tyrosine kinase signaling pathway and cell surface receptor signaling pathway), development and multicellular process (positive regulation of multicellular organismal process; positive regulation of developmental process; multicellular organismal process; multicellular organism development; positive regulation of cellular process; and positive regulation of biological process), as well as transcriptional and metabolism (DNA-templated transcription; nuclear-transcribed mRNA catabolic process, no-go decay; RNA biosynthetic process; regulation of DNA-templated transcription; obsolete regulation of nucleic acid-templated transcription; regulation of RNA biosynthetic process; regulation of RNA metabolic process; obsolete aromatic compound biosynthetic process; and regulation of macromolecule metabolic process) in rat DRGs after sciatic nerve injury. Two GO molecular functions related to binding, namely, Protein binding and Binding, were also among the most significantly enriched GO terms in the differentially expressed mRNAs in rat DRGs after sciatic nerve injury (Figure 1C). Different from injured DRGs following sciatic nerve injury, the most significantly enriched GO terms in dorsal root injury-induced differentially expressed mRNAs were predominantly related to molecular functions and cellular components. Significantly involved molecular functions were binding-related functions (CCR2 chemokine receptor binding; protein binding; and binding) and enzyme activity-related functions (UDP-glycosyltransferase activity and transferase activity) while significantly involved cellular components were mainly chromatin silencing complex, ubiquitin ligase complexes, vesicle-related structures, and neuron-related structures (Figure 1C).

KEGG pathway analysis demonstrated that the most significantly involved KEGG pathway in rat DRGs after sciatic nerve injury was metabolic pathway Lysine degradation. Other top enriched KEGG pathways were primarily associated with signal transduction (MAPK signaling pathway, TNF signaling pathway, TGF-β signaling pathway, Jak-STAT signaling pathway, and cAMP signaling pathway), signaling molecules and interaction (cytokine-cytokine receptor interaction), cellular process/cell growth and death (p53 signaling pathway, signaling pathways regulating pluripotency of stem cells, and cellular senescence), and the immune system (Toll-like receptor signaling pathway, IL-17 signaling pathway, and complement and coagulation cascades) (Figure 1D). In contrast, dorsal root injury predominantly activated disease-associated pathways in rat DRGs while sharing common signaling mechanisms with sciatic nerve injury, particularly in MAPK signaling pathway, TNF signaling pathway, cytokine-cytokine receptor interaction, cellular senescence, IL-17 signaling pathway (Figure 1D).

### PTPRG exhibits opposite expression changes in DRGs following sciatic nerve injury and dorsal root injury

**FIGURE 2:**
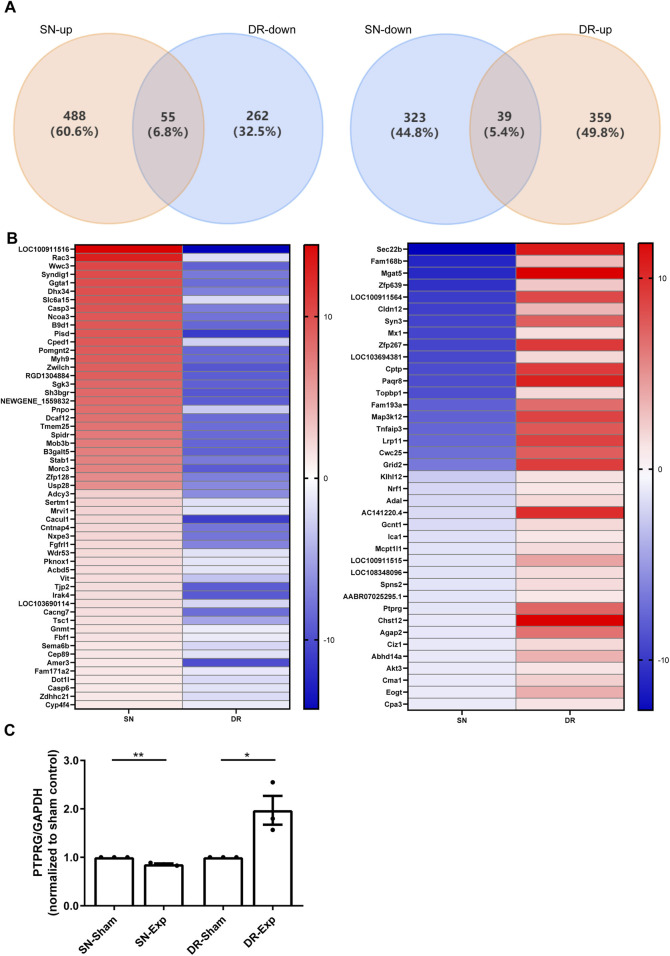
Identification of mRNAs with inverse expression patterns in rat DRGs following sciatic nerve injury and dorsal root injury. **(A)** Venn diagram of the overlapping differentially expressed mRNAs. **(B)** Heatmap visualization of bidirectionally regulated genes. **(C)** qRT-PCR examination of the relative mRNA expression level of PTPRG in rat DRGs after sciatic nerve injury and dorsal root injury. Unpaired t-test; n = 3 biological independent experiments; *, p-value < 0.05; **, p-value < 0.01.

Bioinformatic analysis demonstrates that sciatic nerve injury and dorsal root injury activate partially overlapping yet mostly distinct transcriptional profiles. We further identified and characterized mRNAs showing opposite expression patterns in rat DRGs between these two diverse injury models. This comparative approach revealed a total of 55 mRNAs that were significantly upregulated in rat DRGs after sciatic nerve injury while downregulated after dorsal root injury, and a total of 39 mRNAs whose expression levels in rat DRGs were reduced after sciatic nerve injury but elevated after dorsal root injury (Figure 2A). And the relative expression levels of these bidirectionally regulated genes were displayed in the heatmap (Figure 2B).

We specifically investigated the expression changes of tumor suppressor genes among these mRNAs demonstrating the opposite regulation patterns and identified PTPRG as a significantly dysregulated candidate. We next validated RNA sequencing explored-dynamic changes of PTPRG expression using qRT-PCR. Our qRT-PCR results corroborated transcriptomic data and demonstrated that sciatic nerve injury induced a significant reduction in PTPRG mRNA levels in DRGs relative to sciatic nerve sham surgery while dorsal root injury resulted in a substantial elevation of PTPRG relative to dorsal root sham surgery (Figure 2C). These qRT-PCR observations provide independent confirmation of the RNA sequencing results, reveal an intriguing bidirectional modulation pattern of this tumor suppressor gene in rat DRGs in response to injury to peripheral and central axonal branches, and reflect that the contrasting expression of PTPRG in DRGs may contribute to distinct regenerative capacities of injured peripheral and central axonal branches.

### PTPRG silencing does not significantly affect neuron apoptosis

**FIGURE 3:**
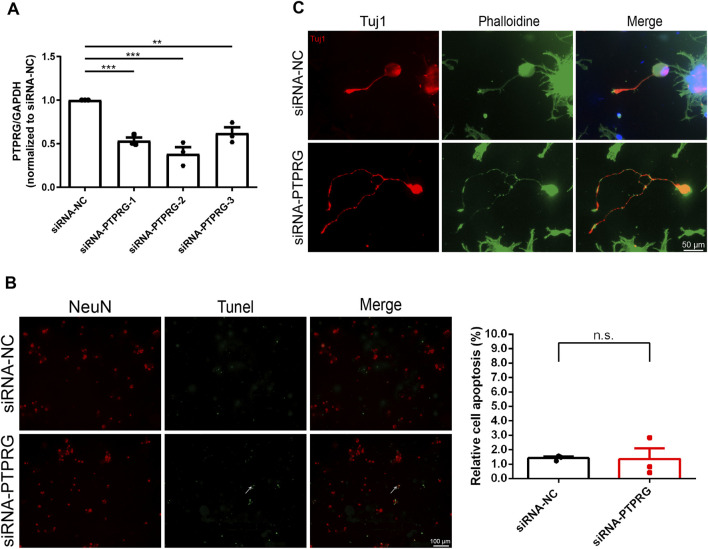
Reduced PTPRG in DRG neurons enhances neurite outgrowth without affecting neuronal survival. **(A)** qRT-PCR examination of the relative mRNA expression level of PTPRG in cultured DRG neurons. Ordinary one-way ANOVA followed by Dunnett’s multiple comparisons test; n = 3 biological independent experiments; **, p-value < 0.01; ***, p-value < 0.001. **(B)** TUNEL staining images of DRG neurons transfected with control siRNA (siRNA-NC) or siRNA targeting PTPRG (siRNA-PTPRG) and quantitative analyses of neuronal apoptosis. Red color indicates NeuN staining and green color indicates TUNEL staining. Arrow indicates representative TUNEL-positive neuron. Scale bar, 100 μm. Unpaired t-test; n = 3 biological independent experiments. **(C)** Tuj1 and phalloidin staining images of DRG neurons transfected with control siRNA or siRNA targeting PTPRG. Red color indicates Tuj1 staining and green color indicates phalloidin staining. Scale bar, 50 μm.

We next explored the biological roles of PTPRG in DRG neurons by performing targeted gene knockdown. Three specific siRNA segments against PTPRG were transfected into cultured rat DRG neurons, all achieving significant knockdown efficiency. The most effective siRNA segment, siRNA-PTPRG-2, reduced PTPRG expression to 38.3% of the control level and was thus applied to subsequent functional studies (Figure 3A). The TUNEL assay revealed low apoptotic activity in both DRG neurons transfected with control siRNA or siRNA against PTPRG, with no significant difference in apoptosis rates observed between siRNA-PTPRG-2-treated DRG neurons and controls (Figure 3B). These observations demonstrate that PTPRG knockdown does not induce cytotoxic effects or alter neuronal survival. We dual-labeled transfected DRG neurons with the neuronal marker Tuj1 and phalloidin. Tuj1 staining outlined the complete neuronal architecture including neuronal somata and extending neurites and hence indicated the successful isolation and maintenance of neurons. Phalloidin staining visualized cytoskeletal F-actin and showed cytoskeletal reorganization during cellular growth. Both immunostaining with neuron-specific antibody Tuj1 and phalloidin labeled F-actin demonstrated enhanced neurite elongation from neuronal soma after PTPRG silencing, indicating the promoting role of PTPRG knockdown in neurite growth (Figure 3C).

### PTPRG silencing promotes neurite outgrowth

**FIGURE 4:**
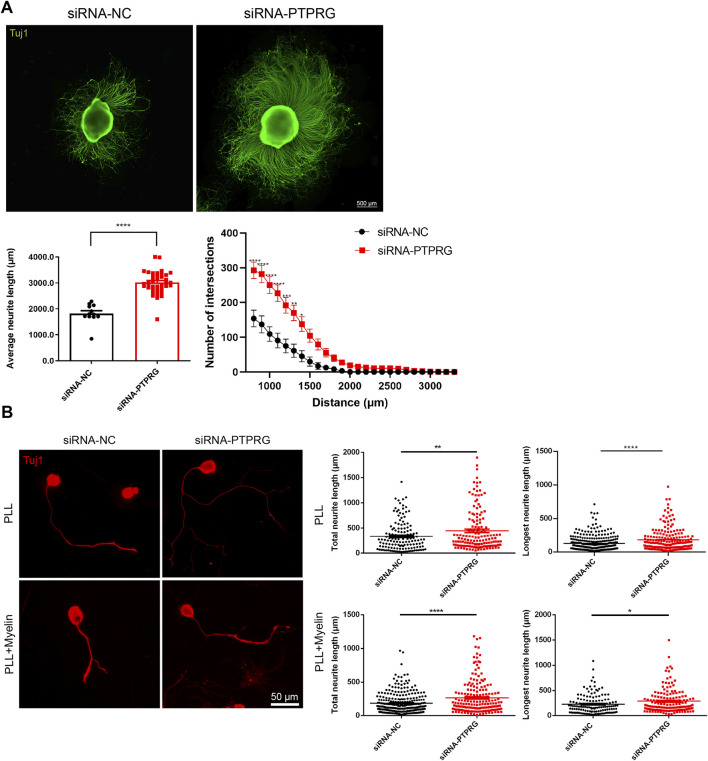
Reduced PTPRG promotes neurite growth and extension. **(A)** Tuj1 staining images of DRG explants transfected with control siRNA or siRNA targeting PTPRG, and quantitative analysis of neurite extension. Scale bar, 500 μm. (Left) The average maximum outgrowth distance measured from the explant edge to the axon tip in four directions per explant. Unpaired t-test; n > 10 DRG explants; ****, p-value < 0.0001. (Right) The length of neurite extension at varying distances from the explant body. Two-way ANOVA following by Du’s multiple comparisons test; n > 10 DRG explants; *, p-value < 0.05; **, p-value < 0.01; ***, p-value < 0.001, ****, p-value < 0.0001. **(B)** Tuj1 staining images of DRG neurons cultured on glass coverslips pre-coated with PLL or PLL plus myelin and quantitative analyses of neurites. Scale bar, 50 μm. Unpaired t-test; n > 130 neurons for neurons cultured on PLL and n > 180 neurons for neurons cultured on PLL plus myelin; *, p-value < 0.05; **, p-value < 0.01; ****, p-value < 0.0001.

Next, we observed the impact of reduced PTPRG on neurite network growth in neonatal rat DRG explants. We transfected freshly isolated neonatal rat DRG explants that were adhered to PLL-coated glass coverslips with PTPRG-targeting siRNA and cultured for 60 h followed by fixation and Tuj1 immunostaining. Immunolabelling of DRG neurites showed that PTPRG knockdown largely boosted the extension of neurites and increased their length and density. We quantified the average outgrowth distance by measuring the vector from the explant edge to the axon tip in four directions per explant. Consistent with the representative images, quantitative analysis showed that the average outgrowth distance significantly increased from 1,820 µm in the siRNA control group to approximately 3,000 µm following PTPRG knockdown (Figure 4A). Additionally, using Sholl analysis of neurite “number of intersections”, we draw concentric circles at 100 µm radial intervals centered on the DRG explants from the DRG explant center and counted the number of intersections between neurites and the concentric circles. Given that the density of neurites near the DRG explant center was too dense for accurate quantification, the numbers of intersections at 800 µm from the DRG explant center to 3,300 µm were recorded. In the siRNA control group, the average number of intersections at 800 µm was around 150. A progressive decline in neurite intersection was observed at longer distance from the center of the DRG explant, with counts decreasing to fewer than 10 at 1,800 µm. Transfection with PTPRG-targeting siRNA resulted in consistently higher numerical values across all measured positions when compared with the control group, with the most pronounced difference observed within 1,400 µm from the DRG explant center. Notably, intersections remained above 10 even at 2,500 µm from the explant center in the PTPRG-knockdown group (Figure 4A). These observations fully reveal the positive effect of reduced PTPRG on neurite outgrowth from DRG explants.

Consistent with DRG explant observations, immunostaining visualization of isolated DRG neurons demonstrated the functional enhancement of neurite outgrowth upon PTPRG suppression. Quantitative analysis revealed that siRNA control-treated DRG neurons exhibited a total neurite length of 335.6 µm and a maximum neurite extension of 222.8 µm as mean values. DRG neurons in the PTPRG siRNA transfected group displayed substantially increased neurite length relative to the control group, with an average total neurite length of 447.5 µm and an average longest neurite length of 285.9 µm (Figure 4B). In addition to the observations of neuronal growth conditions on PLL-coated substrates, we also detected the growth conditions of DRG neurons cultured on substrates coated with PLL plus myelin, a major inhibitory factor for neurite growth. DRG neurons in both control and PTPRG siRNA-transfected groups exhibited attenuated neurite lengths in myelin-containing inhibitory microenvironments, reflecting the inhibitory influence of myelin substrates on neurite extension. Notably, siRNA-mediated PTPRG suppression induced an approximately 1.5-fold enhancement in both total length and longest length of neurites relative to the control group, demonstrating that PTPRG silencing could partially overcome the inhibitory effects of myelin (Figure 4B).

### PTPRG silencing promotes axonal regeneration

**FIGURE 5:**
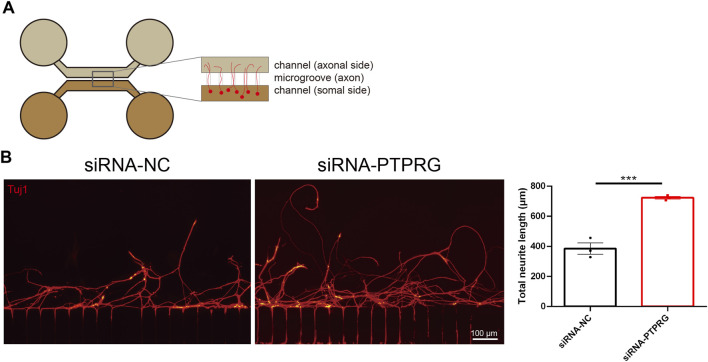
Reduced PTPRG promotes axonal regeneration. **(A)** Schematic diagram of the experimental model for analyzing axonal regeneration following *in vitro* axotomy using a microfluidic device. **(B)** Tuj1 staining images of regenerated axons of DRG neurons transfected with control siRNA or siRNA targeting PTPRG and quantitative analysis of regenerated axon length. Scale bar, 100 μm. Unpaired t-test; n = 3 biological independent experiments; ***, p-value < 0.001.

Having determined the promoting effects of PTPRG downregulation on neurite growth and extension, we next explored the functional roles of PTPRG in axonal regeneration. We employed a two-compartment microfluidic chamber and established a controlled *in vitro* axotomy model that allows precise transection of neuronal axons within the axonal compartment without directly damaging neuronal somata (Figure 5A). PTPRG-silenced DRG neurons demonstrated increased numbers of regenerated axons and greater axon length after axonal injury and subsequent regrowth. Axonal regeneration was quantified by measuring the average length of the ten longest regenerating axons per batch of injured neurons across three independent experimental replicates. Quantitative analysis showed that PTPRG-silenced neurons exhibited an approximately 1.9-fold potentiation of axonal regenerative capacity, achieving significantly elongated regenerated axons with an average length of 723.2 µm compared to the control group with an average length of 385.3 µm (Figure 5B).

### PTPRG silencing is associated with changes in metabolism and the elevation of transcription factor-coding gene prospero-related homeobox 1 (PROX1)

**FIGURE 6:**
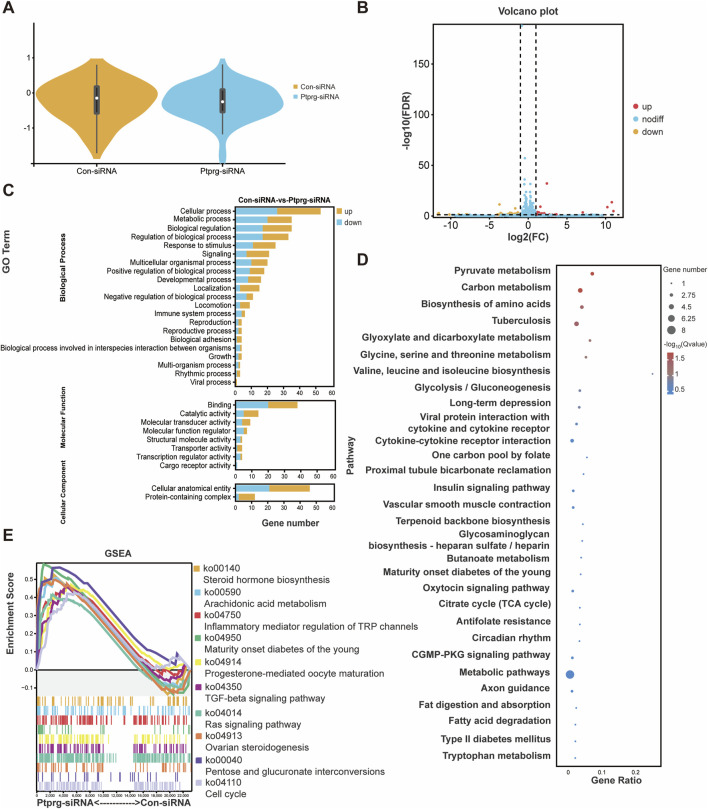
Reduced PTPRG induces genetic changes in DRG neurons. **(A)** The expressions of mRNAs in DRG neurons transfected with control siRNA or siRNA targeting PTPRG. **(B)** Volcano plot of differentially expressed mRNAs. Red color indicates upregulation and blue color indicates downregulation. **(C)** Enriched GO terms. **(D)** Enriched KEGG pathways. **(E)** GSEA of top involved KEGG pathways ranked by positive NES scores.

To explore the potential mechanisms associated with PTPRG silencing, we performed sequencing analysis. Using violin plots to visualize gene expression from sequencing results, we found that neurons transfected with control siRNA and those transfected with PTPRG siRNA exhibited comparable levels of gene expression (Figure 6A). We identified a total of 83 differentially expressed mRNAs, comprising 42 upregulated and 41 downregulated mRNAs (Figure 6B). Top enriched GO biological process terms with more than 30 annotated differentially expressed genes were GO biological process terms cellular process, metabolic process, biological regulation, and regulation of biological process, GO molecular function term binding, as well as GO cellular component term cellular anatomical entity (Figure 6C). Many metabolism-related pathways were identified to be top enriched KEGG pathways, including pyruvate metabolism, carbon metabolism, biosynthesis of amino acids, glyoxylate and dicarboxylate metabolism, glycine, serine and threonine metabolism, valine, leucine and isoleucine biosynthesis, and glycolysis/gluconeogenesis, one carbon pool by folate, terpenoid backbone biosynthesis, glycosaminoglycan biosynthesis-heparan sulfate/heparin, butanoate metabolism, citrate cycle (TCA cycle), metabolic pathways, fatty acid degradation, and tryptophan metabolism (Figure 6D). In addition to analyzing significant changes in individual genes, we also explored the coordinated changes across gene sets using GSEA and directly visualized expression trends genome-wide. Consistent with the enriched metabolic processes in differentially expressed genes, many enriched GSEA pathways with high normalized enrichment score (NES) values were associated with biosynthesis and metabolism, such as steroid hormone biosynthesis, arachidonic acid metabolism, and maturity onset diabetes of the young (Figure 6E).

**FIGURE 7:**
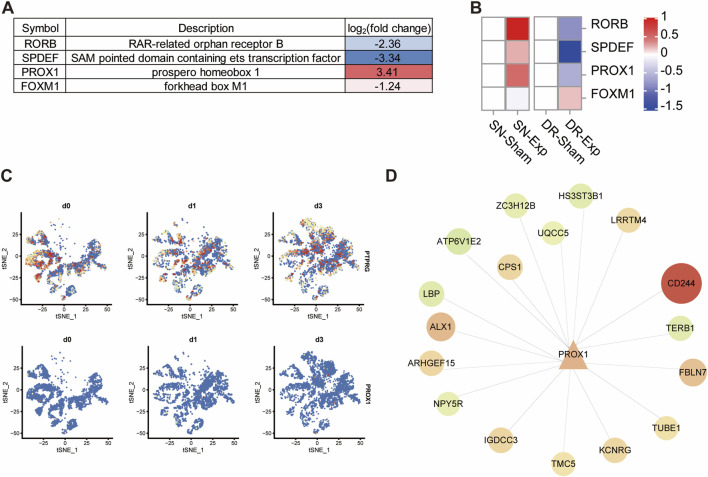
Reduced PTPRG increases the expression of transcription factor coding gene PROX1. **(A)** List of differentially expressed transcription factor coding genes in DRG neurons transfected with control siRNA or siRNA targeting PTPRG. **(B)** Expressions of SPDEF, PROX1, RORB, and FOXM1 in rat DRGs after sciatic nerve injury, dorsal root injury, or corresponding sham surgery. **(C)** tSNE distribution of PTPRG and PROX1 in mouse DRG neurons at 0, 1, and 3 days after sciatic nerve injury. **(D)** Genetic network of PROX1 and its downstream target genes. Upregulated genes in DRG neurons transfected with siRNA targeting PTPRG were labelled with red color while downregulated genes were labelled with green color.

Following PTPRG silencing in DRG neurons, four transcription factor-encoding mRNAs, including SPDEF (SAM pointed domain containing ETS transcription factor), PROX1 (prospero-related homeobox 1), RORB (RAR related orphan receptor B), and FOXM1 (forkhead box M1), were altered among the 83 differentially expressed mRNAs. SPDEF, RORB, and FOXM1 were significantly downregulated after PTPRG knockdown while PROX1 was robustly upregulated (Figure 7A). Next, we characterized the dynamic expression patterns of these transcription factor-encoding mRNAs in injured rat DRGs. The expression of PROX1 was upregulated following sciatic nerve injury but downregulated following dorsal root injury, which was inversely associated with the expression pattern of PTPRG (Figure 7B). The dynamic temporal distribution patterns of PTPRG and PROX1 in DRG neurons from single-cell RNA sequencing data showed that consistent with our bulk sequencing outcomes, PTPRG expression was decreased in DRG neurons following sciatic nerve injury, whereas PROX1 expression was increased (Figure 7C). The observed inverse association of PTPRG and PROX1 in both PTPRG knockdown and distinct nerve injury models leads us to speculate that PTPRG might function as a negative regulator of PROX1. Systematic analysis of PROX1 and its potential downstream targets suggested that PROX1 is potentially associated with the upregulation of CD244, TUBE1, FBLN7, KCNRG, TMC5, IGDCC3, ARHGEF15, ALX1, CPS1, and LRRTM4 as well as the downregulation of TKTL1, TERB1, NPY5R, LBP, ATP6V1E2, ZC3H12B, UQCC5, and HS3ST3B1 (Figure 7D).

## Discussion

The divergent regenerative responses of DRG peripheral versus central axonal branches serve as a powerful model to dissect the fundamental mechanisms underlying successful versus failed axonal regeneration. To our knowledge, a comparative analysis of mRNA expression changes in rat DRGs following injury to either peripheral or central axonal branches has not been previously reported, despite the rat being one of the most frequently used animal model in nerve injury research (Geuna, 2015). In the current study, our characterization of the transcriptional profiles in rat L4-L5 DRGs at 1 day post-axotomy of sciatic nerves or dorsal roots demonstrates both conserved and injury-specific molecular changes and associated biological activities in rat DRGs after acute peripheral and central axonal injuries.

Bioinformatic analysis of differentially expressed mRNAs in rat DRGs reveals that dorsal root injury shares overlapping signaling cascades with sciatic nerve injury, particularly in top enriched MAPK signaling, TNF signaling, and IL-17 signaling. MAPK cascades are well established as early sensors in response to nerve injury and key regulators of axonal regeneration (Hammarlund et al., 2009; Nix et al., 2011). Recent studies have elucidated the engagement of TNF signaling and IL-17 signaling in the regulation of nerve injury responses and regenerative progression. The inhibition of TNF-α has been shown to compromise axon regeneration, indicating the promoting role of pro-inflammatory cytokine TNF-α in axonal elongation and nerve repair (Tsarouchas et al., 2018). IL-17, another inflammatory cytokine, also exerts crucial pro-regenerative effects. IL-17 cytokine, secreted by commensal Th17 cells generated through adaptive immunity, binds to IL-17 receptor on neurons and largely accelerates axonal regrowth and local nerve regeneration (Enamorado et al., 2023). In addition to MAPK signaling, TNF signaling, and IL-17 signaling, cellular senescence is also enriched in differentially expressed mRNAs in rat DRGs after both injuries to sciatic nerve and dorsal root. Cellular senescence is not only recognized as a hallmark of aging but also as a critical process for injury response, tissue remodeling, and wound healing. Our previous study has demonstrated obvious alterations in the expression of cellular senescence-related genes in rat DRGs following sciatic nerve injury (Shen et al., 2022). In the current study, by leveraging transcriptomic data and KEGG pathway analysis, we show that cellular senescence is activated in DRGs not only after peripheral nerve injury but also following central nerve injury, underscoring the critical role of cellular senescence in axonal regeneration.

We also identified several specific KEGG pathways specifically enriched after sciatic nerve injury, including the TGF-β signaling pathway, Jak-STAT signaling pathway, cAMP signaling pathway, and p53 signaling pathway. Notably, emerging studies have reported that these signaling cascades, which are often found to be differentially expressed after injury, play important roles in enhancing neurite outgrowth and axonal regeneration (Ma et al., 2019; Vidal et al., 2013; Vigneswara et al., 2014; Elsaeidi et al., 2014; Li et al., 2015; Di Giovanni and Rathore, 2012; Zhang F. M. et al., 2023). Hence, the selective activation of these signaling pathways in rat DRGs after sciatic nerve injury likely underlies the regenerative advantage of injured peripheral axonal branches. Therapeutic targeting of these pathways may thus represent a promising therapeutic strategy to enhance neuronal intrinsic regenerative capacity. The differences in enriched GO terms between sciatic nerve injury and dorsal root injury also reveal fundamentally distinct injury responses. Following sciatic nerve injury, the dominant enrichment of biosynthetic and metabolic processes. On the contrary, DRGs after dorsal root injury show prominent involvement of chromatin silencing complex, a GO cellular component term closely associated with transcriptional repression. The metabolic activation after sciatic nerve injury versus transcriptional silencing after dorsal root injury provides a compelling molecular explanation for the divergent regenerative capacities of DRG neurons after injury to their peripheral or central axonal branches. The anabolic state in DRG neurons after peripheral axonal branch injury fuels successful axonal regrowth, whereas the repressive chromatin landscape after central axonal branch injury generates a barrier to regeneration.

In addition to the evaluation of involved biological activities in rat DRGs after regeneration-permissive peripheral axonal injury and regeneration-incompetent central axonal injury, we systematically identified candidate regulators of axonal regeneration by examining gene expression patterns. Genes downregulated in rat DRGs after peripheral axonal injuries but upregulated in central axonal injuries represent potential axonal regeneration inhibitors, while those showing the opposite expression trend represent potential axonal regeneration promoters. Among these potential axonal regeneration regulators, PTPRG emerges as a compelling target, given the well-explored functions of tumor suppressor genes in intrinsic axonal growth capacity (Krishnan et al., 2016; Oh et al., 2018). The injury-induced downregulation of PTPRG in DRGs following sciatic nerve injury may thus contribute to the regeneration-permissive characteristic of peripheral axonal branches, while its upregulation following dorsal root injury may participate in the regeneration-inhibitory characteristic of central axonal branches.

PTPRG demonstrates ubiquitous expression across mammalian tissues, while manifesting significant downregulation in various malignancies, a characteristic indicative of its tumor suppressor function (Boni and Sorio, 2021). In the mammalian nervous system, PTPRG is expressed in both embryonic and adult neurons (Lamprianou et al., 2006). Although deletion of PTPRG does not impair nervous system development, dysregulation of PTPRG and/or PTPRG variants has been associated with multiple neurological disorders, including neuroinflammation, schizophrenia, and Alzheimer’s disease, implying that PTPRG may participate in the regulation of neuronal activities (Lamprianou et al., 2006; Lorenzetto et al., 2014; Boni et al., 2022; Cressant et al., 2017). While emerging evidence suggests the involvement of PTPRG in pathobiology of the nervous system, its biological functions in axonal regeneration remains incompletely characterized. Early experimental evidence from PTPRG-transfected PC12D cells has revealed that elevated expression of PTPRG remarkably suppresses nerve growth factor-induced neurite outgrowth (Shintani et al., 2001). A recent study demonstrates that binding of chondroitin sulfate to PTPRG induces the activation of PTPRG, mediates the disruption of autophagy flux, leads to the formation of dystrophic endball, and subsequently inhibits axonal regeneration (Sakamoto et al., 2019). These observations suggest that PTPRG may function as a critical mediator of extracellular stimulating or inhibitory molecules in axonal growth and elongation. Still, a critical knowledge gap persists regarding the intrinsic regulatory function of PTPRG in axonal regenerative capacity. In our current study, TUNEL assay analysis demonstrated that reduced PTPRG expression does not significantly influence neuronal apoptosis. Using an DRG explant culture model and a neuronal culture model, we investigated the role of PTPRG in neurite dynamics and found that PTPRG silencing robustly enhances neurite outgrowth, even in the presence of myelin-associated inhibitory factor. Moreover, using a microfluidic device-based *in vitro* axonal regeneration model, we demonstrated that PTPRG knockdown supports the elongation of injured axons. These observations fully indicate that tumor suppressor gene PTPRG acts as a critical restricting factor for axonal growth and suggest that PTPRG silencing serves a valuable therapeutic strategy for accelerating nerve regeneration.

The potential underlying mechanisms of enhanced growth capacity driven by PTPRG knockdown were investigated using RNA sequencing. Bioinformatic analysis of differentially expressed genes in cultured DRG neurons after PTPRG knockdown revealed that PTPRG is associated with cellular metabolism and biosynthesis. The observed activation of cellular metabolism and biosynthesis in DRG neurons following PTPRG knockdown aligns with the prominent role of metabolic processes in injured DRGs during regeneration-permissive sciatic nerve injury. Metabolic processes supply precursor molecules and energy for biosynthesis and critically govern cell fate under multiple physiological and pathological conditions, such as inflammation, cancer, and tissue regeneration (Feng et al., 2025). Numerous metabolic pathways undergo dynamic changes following nerve injury (Zhang et al., 2022). Increased production of triglyceride storage lipids following axotomy is associated with regeneration failure, while switching triglyceride synthesis to phospholipid membrane lipid synthesis via depleting neuronal lipin 1 or diglyceride acyltransferases largely contributes to axonal regeneration (Yang et al., 2020; Chen et al., 2024). In addition to lipid metabolism, glucose uptake and glycolysis are also essential for the normal function of neurons (Li et al., 2023). Reprogramming of glucose metabolism via modulation of the pyruvate dehydrogenase beta subunit alters cellular energy production and regulates axonal regeneration (Jiang et al., 2023). Although PTPRG is recognized as a key regulator in cancer, its role in cancer-associated metabolic reprogramming remains poorly understood. Herein, we report the involvement of PTPRG in neuronal metabolic regulation, meaningfully advancing our understanding of the biological functions of PTPRG.

PTPRG-mediated differentially expressed transcription factor-coding genes were further explored considering the essential roles of transcriptional factors in nerve regeneration (Zhang Y. et al., 2023). PROX1, a member of the homeobox transcription factor family with both oncogenic and tumor suppressive characteristics (Elsir et al., 2012), exhibited increased gene expression after PTPRG silencing. Moreover, in rat DRGs, PROX1 showed reciprocal expression dynamics to PTPRG following injuries to different branches of DRG neurons: PROX1 level rose after sciatic nerve injury where PTPRG was downregulated but declined after dorsal root injury where PTPRG was upregulated. Consistent with bulk sequencing data, single-cell sequencing revealed the decreased expression of PTPRG and the increased expression of PROX1 in DRG neurons following injury to mouse sciatic nerves. PROX1 has been reported to be essential for neurogenesis during nervous system development whereas its effect on nerve regeneration has not been fully explored (Kaltezioti et al., 2010; Holzmann et al., 2015). Building on evidence that PROX1 is associated with metabolic reprogramming (Gan et al., 2023; Meçe et al., 2022; Alfaro et al., 2023), we hypothesize that PTPRG may modulate cellular metabolic pathways to influence axonal regenerative capacity through its negative regulation of PROX1. Notably, a key mechanism by which PROX1 regulates energy metabolism involves its ability to activate mTOR signaling (Wang et al., 2022). PTPRG has been reported to modulate mTOR signaling by suppressing Akt, a critical upstream kinase in the PI3K/Akt/mTOR pathway (Cheung et al., 2015). The PI3K/Akt/mTOR pathway, a central regulator of metabolic reprogramming and a well-established driver of axonal regeneration (Park et al., 2010), may therefore represent a potential link between PTPRG, PROX1, and axonal regeneration. We speculate that downregulated PTPRG in DRGs after peripheral axonal branch injury may contribute to the activation of Akt/mTOR and indirectly promote mTOR signaling by elevating PROX1 expression. These synergistic actions may orchestrate metabolic reprogramming to fuel the energetic and biosynthetic demands of regenerating axons. Conversely, upregulated PTPRG in DRGs after central axonal branch injury may constrain mTOR activity and hinder axonal regeneration.

Transcription factors orchestrate the molecular program in multiple physiological and pathological conditions, including nerve repair and regeneration, by recognizing and binding to specific DNA sequences in regulatory regions. The transcription factor PROX1 exhibits dual transcriptional regulatory activity, functioning as both an activator and repressor of its target genes. For instance, in fast skeletal muscle, elevated expression of PROX1 induces the upregulation of a series of slow muscle fibre specific genes but leads to the downregulation of fast MyHC genes such as Myh2 and Myh4 while genetic ablation of PROX1 induces opposite transcriptional outcomes (Kivelä et al., 2016). Herein, through integrated analysis of transcription factor databases including knockTF, FIMO, and JASPAR, we predicted many potential downstream regulatory targets of PROX1. The biological functions of PROX1 as well as its downstream targets in nerve regeneration remain unexplored. Our current findings indicate that not only PTPRG, but also PTPRG-regulated transcription factor PROX1 as well as its associated molecular network may represent novel regulatory elements in the regeneration process following nerve injury.

Taken together, our current study, by comparing gene profiling of L4-L5 rat DRGs at 24 h post sciatic nerve injury and dorsal root injury, delineates distinct characteristics underlying the divergent regenerative outcomes between peripheral axonal branch and central axonal branch axotomy models and identify tumor suppressor PTPRG as a potent negative regulator of neurite growth and axonal regeneration. These findings extend our understanding of regulatory elements governing neuronal regenerative competence and establish PTPRG as a novel therapeutic target for promoting nerve regeneration.

## Data Availability

The datasets presented in this study can be found in online repositories. The names of the repository/repositories and accession number(s) can be found in the article/supplementary material.
